# Complete Genome Sequence of *Streptomyces* Siphophage Sycamore

**DOI:** 10.1128/MRA.01343-20

**Published:** 2021-01-07

**Authors:** Xing-Han Zhang, Alejandra Marquez, James Clark, Isla Hernandez, Madeline Rivera, Mei Liu, Ben Burrowes

**Affiliations:** a Department of Biochemistry and Biophysics, Texas A&M University, College Station, Texas, USA; b Center for Phage Technology, Texas A&M University, College Station, Texas, USA; Portland State University

## Abstract

*Streptomyces* sp. strain Mg1 is a competitive soil-dwelling bacterium that secretes antibiotics that inhibit growth of Bacillus subtilis. Here, we present the genome sequence of Sycamore, a 44,694-bp *Streptomyces* sp. Mg1 siphophage with 66 predicted protein-coding genes, that is similar to phage genome sequences in the *Lomovskayavirus* genus.

## ANNOUNCEMENT

*Streptomyces* spp. are Gram-positive soil bacteria. Like other streptomycetes, *Streptomyces* sp. strain Mg1 secretes numerous antibiotics that offer the bacterium a growth advantage in the soil environment (1, 2), such as the ability to degrade colonies of Bacillus subtilis (2). Here, we describe the isolation and genome annotation of *Streptomyces* sp. Mg1 siphophage Sycamore.

Bacteriophage Sycamore was isolated in February 2019 from an Illinois topsoil sample by plaque purification on *Streptomyces* sp. Mg1 (provided by Paul Straight, Texas A&M University) grown at 30°C on nutrient agar or broth supplemented with 10 mM MgCl_2_, 8 mM Ca(NO_3_)_2_, and 0.5% glucose using previously reported methods (3). To determine phage morphology, crude Sycamore lysates were stained with 2% (wt/vol) uranyl acetate and viewed via transmission electron microscopy (data not shown) at the Texas A&M Microscopy and Imaging Center (4). DNA was purified as previously described (3) using DNA Wizard DNA clean-up kits and then prepared as Illumina libraries using a Nextera Flex kit to be sequenced on an Illumina MiSeq instrument with paired-end 300-bp reads using V2 500-cycle chemistry. Sequence reads were quality controlled with FastQC (www.bioinformatics.babraham.ac.uk/projects/fastqc) and manually trimmed using FastX 0.0.14 (http://hannonlab.cshl.edu/fastx_toolkit/download.html). Using SPAdes v3.5.0, a single contig at 275.4-fold coverage was assembled from 686,406 total sequence reads (5). The contig was PCR amplified off the ends (forward primer, 5′-GTAGTGACCACACCCTAGGTAA-3′; reverse primer, 5′-GTATGAGTCGCTGGTCAACAG-3′), and the product was Sanger sequenced to verify sequence closure. Protein-coding genes were predicted with GLIMMER v3 and MetaGeneAnnotator v1.0, tRNAs with ARAGORN v2.36, and rho-independent termination sites with TransTermHP v2.09 (6–9). Functional gene predictions relied on InterProScan v5.33, TMHMM v2.0, and BLAST v2.9.0 (with a 0.001 maximum expectation value cutoff) against the following databases: NCBI nonredundant, UniProtKB Swiss-Prot, and TrEMBL (10–13) (accessed 23 April 2020). Structural predictions were performed with the HHSuite v3.0 tool HHpred (14). The genome-wide DNA sequence similarity to other phages was calculated using progressiveMauve v2.4 (15). Excluding HHpred, all tools were accessed at the Center for Phage Technology Galaxy interface and run with default parameters, and annotation was performed in Web Apollo (hosted online at https://cpt.tamu.edu/galaxy-pub/) (16–18).

Sycamore has a genome size of 44,694 bp with a G+C content of 63%, which is much lower than the characteristically high G+C content observed in *Streptomyces* species (19). Our analysis predicted 68 protein-coding genes, of which 37 were assigned putative functions, and 7 tRNA genes, yielding an overall 90% coding density. A BLASTp search revealed that Sycamore shares the greatest amino acid identity with phages of the *Lomovskayavirus* genus (taxonomy identification number [taxid] 308912), of which *Streptomyces* phage Attoomi (GenBank accession number NC_047905.1) shared the most protein-coding genes, with 25 similar unique proteins. Interestingly, the predicted lysis cassette of Sycamore lacked a predicted holin, and the putative endolysin *N*-acetylmuramidase and two-component spanin were detected approximately 20 kb apart. Moreover, the sequence of a likely tape measure frameshift varied from the 5′-CGGGGGCG-3′ slippery sequence of phage Mu by a single G/A point mutation at the fourth nucleotide (20). No introns were detected.

### Data availability.

The genome sequence of Sycamore was deposited in GenBank with accession number MT701593.1. The associated BioProject, SRA, and BioSample accession numbers are PRJNA222858, SRR11558337, and SAMN14609635, respectively.
